# Microbial diversity, functional genomics and antibiotic resistance in integrated chicken and fish farming systems of Bangladesh

**DOI:** 10.1371/journal.pone.0344367

**Published:** 2026-04-08

**Authors:** S. M. Kador, Jannatul Ferdus Shila, Sinthea Afrin, Jarin Jannat, Khondoker Tanjim Islam, Rafid Nahian Rubaiyat, Mohammad Imtiaj Uddin Bhuiyan, Tanay Chakrovarty, Md. Shazid Hasan, Najmuj Sakib, M. Shaminur Rahman, Ovinu Kibria Islam, Md. Tanvir Islam

**Affiliations:** 1 Department of Microbiology, Jashore University of Science and Technology, Jashore, Bangladesh; 2 Department of Environmental Health Science, University of Georgia, Athens, Georgia, United States of America; Florida A&M University: Florida Agricultural and Mechanical University, UNITED STATES OF AMERICA

## Abstract

The integrated chicken and fish farming system in Bangladesh is widely practiced for its resource efficiency, yet its microbial structure, functional potential, and associated antimicrobial resistance risks remain poorly understood. This study investigated microbial communities, metabolic functions, and antimicrobial resistance profiles across multiple components of integrated farming systems, including chicken gut, chicken droppings, feed, fish intestine, and pond sediment. Microbial profiling was performed using 16S ribosomal ribonucleic acid (rRNA) gene sequencing, functional metagenomic prediction, and culture-based isolation, complemented by antimicrobial susceptibility testing. A total of 2,838 operational taxonomic units were identified, with bacteria constituting the vast majority of detected microorganisms. Microbial community composition was strongly shaped by sample type, reflecting distinct ecological niches within the farming system. Chicken gut samples were dominated by Firmicutes, feed samples by Cyanobacteria, and sediment samples exhibited the highest microbial diversity, including taxa involved in biogeochemical cycling. Functional analysis revealed that pathways related to amino acid and carbohydrate metabolism were most abundant across all samples, while sediment and feed were enriched in pathways associated with xenobiotic degradation, suggesting a role in environmental detoxification. Culture-based methods isolated clinically relevant bacteria, including *Escherichia coli* and *Proteus mirabilis*, although metagenomic analysis indicated that these organisms represented only a minor fraction of the overall microbial community. Antimicrobial susceptibility testing demonstrated notable resistance, particularly to tetracyclines and fluoroquinolones. Metagenomic analysis further identified multiple antimicrobial resistance genes, with several showing strong associations with specific bacterial genera. This study provides the first comprehensive characterization of microbial diversity, functional capacity, and antimicrobial resistance within integrated chicken and fish farming systems in Bangladesh, highlighting potential environmental reservoirs of resistance and underscoring the need for improved management strategies to enhance sustainability and reduce public health risks.

## 1. Introduction

The Integrated Chicken and Fish Farming (ICFF) system is a sustainable agricultural practice that integrates aquaculture with poultry production to enhance resource efficiency and economic viability [1]. This farming approach is widely adopted in Bangladesh due to its low input requirements and its capacity to recycle nutrients across interconnected production compartments [2]. The close physical and biological integration of livestock, aquatic environments, and sediments gives rise to complex ecological interactions that sustain diverse microbial communities, which are fundamental to maintaining productivity, nutrient balance, and environmental integrity within these systems [3]. Microbial communities in integrated farming environments mediate key ecological processes, including nutrient cycling, organic matter degradation, and disease regulation, thereby exerting a direct influence on animal health and system sustainability [4,5]. In poultry, gut-associated microbial taxa—particularly members of the phylum Firmicutes—contribute to fermentation efficiency and nutrient assimilation [6,7]. In contrast, aquatic compartments typically harbor diverse bacterial assemblages dominated by Proteobacteria and Bacteroidetes, which play essential roles in regulating water quality and supporting fish health [8,9]. Despite the recognized importance of these microbial functions, comprehensive characterization of microbial diversity and functional potential across the interconnected components of ICFF systems remains limited, particularly in low- and middle-income countries such as Bangladesh.

Recent advances in functional genomics, including high-throughput 16S ribosomal ribonucleic acid gene sequencing and metagenomic pathway inference, have substantially improved our ability to resolve microbial community composition and metabolic capacity in complex ecosystems [10–12]. These approaches offer critical insights into microbially mediated processes such as nitrogen transformation and the persistence and dissemination of antibiotic resistance genes. The latter has emerged as a major global concern, as extensive antibiotic use in agricultural systems has accelerated the development and spread of antimicrobial resistance, posing significant risks to animal, environmental, and public health [13–15]. Resistance determinants such as *blaTEM*, *tetM*, and *sul1* are known to persist within agricultural microbiomes, with animal gastrointestinal tracts and sediments functioning as important reservoirs and facilitating horizontal gene transfer through mobile genetic elements [16–18]. Our previous investigation of integrated chicken and fish farming systems in Bangladesh revealed a substantial burden of antibiotic resistance genes, with tetracycline resistance genes predominating and farm-associated droppings and sediments identified as major hotspots for resistance accumulation and exchange [19]. More broadly, these observations underscore growing concerns that integrated farming systems by directly linking animal, aquatic, and sedimentary compartments, may create favorable conditions for the persistence and circulation of antimicrobial resistance across environmental niches. A comprehensive, system-level evaluation of microbial diversity, functional potential, and resistance dynamics is therefore essential to elucidate how these interconnected environments collectively shape microbial ecology and influence antimicrobial resistance dissemination.

In this context, the present study provides the first comprehensive analysis integrating microbial community profiling, functional genomic assessment, and phenotypic antimicrobial resistance evaluation within an integrated chicken and fish farming system in Bangladesh. By concurrently examining poultry-associated, aquatic, and sediment compartments, this work characterizes patterns of microbial diversity, metabolic potential, and antibiotic resistance distribution, as well as their associations with clinically relevant bacterial pathogens. By addressing critical gaps in current knowledge, the study offers evidence-based insights to inform sustainable farm management practices, strengthen antibiotic stewardship, and mitigate antimicrobial resistance risks in integrated farming systems [19–21].

## 2. Methodology

### 2.1. Ethical approval

The study protocol received ethical approval from the Ethical Review Committee of the Faculty of Biological Sciences at Jashore University of Science and Technology, Jashore, Bangladesh (Approval No.: ERC/FBST/JUST/2024–200) (S1 Data). All experimental procedures strictly followed international standards including: (a) the ARRIVE guidelines (Animal Research: Reporting of In Vivo Experiments) [22] and (b) the American Veterinary Medical Association (AVMA) Guidelines for the Euthanasia of Animals: 2020 Edition [23]. We implemented multiple measures to ensure animal welfare, including pre-procedural fasting (4 hours for chickens, 24 hours for fish), minimization of handling stress, and immediate processing post-euthanasia to maintain sample integrity. Veterinary supervision was maintained throughout all procedures to verify compliance with ethical standards.

### 2.2. Sample collection and preparation of isolates

Three representative integrated chicken-fish farms in Jashore, Bangladesh (geographic coordinates provided in S1 Table) were selected based on standardized operational criteria including farm size (>0.5 acres), flock size (>100 chicken), and fish stock (>500 fish). From each farm, we systematically collected nine samples encompassing key system components: feed (n = 1, 50g from central feeder), chicken gut (n = 2, duodenum and ileum sections), fresh droppings (n = 2, collected within 30 minutes post-defecation), pond sediment (n = 2, composite samples from 10 cm depth), and fish intestine (n = 2, anterior and posterior sections), yielding a total of 27 samples (Table 1, S1 Data). All animal procedures were performed by a team of three trained researchers, including a PhD-level veterinary doctor. For chicken euthanasia, we employed CO₂ inhalation in a certified euthanasia chamber (VetEquip Model 901806, internal volume: 40 L) following AVMA guidelines. A pre-fill method was used, establishing an initial chamber concentration of 30% CO₂ concentration (certified gas mixture, BOC Bangladesh Ltd.). Birds were then introduced, and the gas flow was adjusted to 28 L/min to achieve 70% displacement per minute. Time to unconsciousness (loss of righting reflex) was 45 ± 12 seconds (mean ± SD, n = 6). Death was confirmed through multiple criteria: absence of corneal reflex, verified cardiac arrest via stethoscope, and fixed dilated pupils, typically within 3–4 minutes of initial exposure. Fish euthanasia was conducted via a gradual fill method in acclimated tanks (80 L) maintained at 26 ± 0.5°C, pH 7.2 ± 0.3, with dissolved oxygen levels of 6.8 ± 0.4 mg/L (measured using a daily-calibrated YSI ProPlus multiparameter meter; Yellow Springs Instruments, Ohio, USA), where CO₂ was introduced at 1.5 L/min through a ceramic diffuser until 10 minutes after opercular movement cessation, which occurred at 5.2 ± 1.1 minutes (mean ± SD, n = 12) post-initiation, followed by pithing for secondary confirmation. Environmental samples were collected using sterile protocols: feed samples were obtained with ethanol-flamed stainless steel scoops into sterile containers, fresh droppings were immediately transferred to anaerobic bags (Oxoid) using disposable spatulas, and pond sediments were sampled at three locations using autoclaved Ekman dredges. All collected samples were placed in sterile Whirl-Pak® bags, maintained on dry ice during transport (<90 minutes), and processed within 4 hours of collection. For long-term preservation, aliquots were stored at −80°C for DNA analysis following established protocols [24]. Complete metadata including precise collection times, handler identification, and euthanasia logs are documented in S1 Data.

**Table 1:** Information regarding the locations of sampling sites and corresponding sample identifiers.

Farms	Locations	Longitude and Latitude	Feed (3)	Chicken gut(6)	Droppings(6)	Sediment (6)	Fish intestine(6)
			DNeasy PowerSoil Pro Kits (Cat. No./ ID: 47014)	QIAamp Fast DNA Stool Mini Kit (Cat. No./ ID: 51604)	QIAamp Fast DNA Stool Mini Kit (Cat. No./ ID: 51604)	DNeasy PowerSoil Pro Kits (Cat. No./ ID: 47014)	QIAamp Fast DNA Stool Mini Kit (Cat. No./ ID: 51604)
			ID	ID	ID	ID	ID
Farm – 1	Solua Bazar, Jashore, Bangladesh	23° 14’ 17.16“N 89° 6’ 5.04“E	I-18	I-16	I-9	I-15	I-19
				I-20	I-10	I-17	I-21
Farm – 2	Jhikargacha, Jashore, Bangladesh	23° 6’ 3.02“N 89° 5’ 58.73“E	I-24	I-3	I-11	I-22	I-1
				I-4	I-12	I-23	I-2
Farm – 3	Khajura, Jashore, Bangladesh	23° 16’ 35.38“N 89° 15’ 13.33“E	I- 25	I-5	I-13	I-26	I-8
				I-7	I-14	I-27	I-6

### 2.3. Microbial isolation and Antibiotic Susceptibility Testing (AST)

For microbial isolation, we employed five selective media: Blood Agar (BA; HiMedia Laboratories, India; Catalog #M108), MacConkey Agar (MAC; Oxoid, UK; Catalog #CM0007B), Xylose Lysine Deoxycholate (XLD) Agar (Merck, Germany; Catalog #1.05287.0500), Mannitol Salt Agar (MSA; Becton Dickinson, USA; Catalog #211443), and Cetrimide Agar (CA; Liofilchem, Italy; Catalog #6104002). Given the documented high prevalence of tetracycline resistance in Bangladeshi livestock systems (Chowdhury et al., 2022), samples were pre-enriched with tetracycline (16 µg/mL, CLSI-recommended breakpoint; Sigma-Aldrich, Catalog #T7660) to selectively isolate resistant strains, yielding 55 isolates distributed across sample types: feed (n = 4), chicken gut (n = 13), droppings (n = 19), fish intestine (n = 9), and sediment (n = 10). Presumptive identification was achieved through biochemical profiling, including catalase (Sigma-Aldrich, Catalog #C1345), oxidase (Becton Dickinson, Catalog #261081), indole production (HiMedia, Catalog #RM287), citrate utilization, and sugar fermentation tests. For the experimental (wet lab) data, counts were based on the number of morphologically and biochemically distinct bacterial isolates obtained from each sample type following growth on selective media with tetracycline enrichment. Identification relied on colony morphology and biochemical tests with each unique species per sample type counted once to avoid redundancy. Percentages were calculated relative to the total isolates (n = 55).

Antibiotic susceptibility testing followed nine antibiotic classes representing commonly used antimicrobials in Bangladesh [25,26], including amoxicillin (AMX, 10 µg; Oxoid, UK; Ref. CT0161B), cefixime (CFM, 5 µg; Ref. CT0653B), imipenem (IPM, 10 µg; Ref. CT0455B), tetracycline (TET, 30 µg; Ref. CT0054B), azithromycin (AZM, 15 µg; Ref. CT0906B), amikacin (AMK, 30 µg; Ref. CT0107B), chloramphenicol (C, 30 µg; Ref. CT0013B), colistin sulfate (COL, 10 µg; Ref. CT0017B), and levofloxacin (LEV, 5 µg; Ref. CT1587B). Testing was conducted using a combination of disk diffusion and agar dilution method, in accordance with Clinical and Laboratory Standards Institute (CLSI) guidelines. Disk diffusion testing was applied to all antibiotics except colistin, while colistin susceptibility was assessed separately using an agar dilution–based approach due to methodological limitations associated with disk diffusion testing for polymyxins. For disk diffusion testing, the Kirby–Bauer method was performed on Mueller–Hinton agar (Oxoid, UK) following CLSI M02 guidelines and antibiotic susceptibility was interpreted based on inhibition zone diameter criteria defined in CLSI M100 (American Society for Microbiology, 2011; CLSI, 2021) [27]. Bacterial suspensions were adjusted to a 0.5 McFarland turbidity standard using a DensiChek instrument (BioMérieux Inc) and inoculated uniformly onto Mueller–Hinton agar plates. Plates were incubated at 37 °C for 24 h, after which inhibition zones were measured in millimeters. Quality control (QC) was performed weekly using *Escherichia coli* ATCC 25922 and *Staphylococcus aureus* ATCC 25923, with observed zone diameters falling within CLSI-specified quality control ranges. Sterile blank disks impregnated with autoclaved distilled water were included as negative controls. Colistin resistance was assessed exclusively using the agar dilution method, in accordance with CLSI recommendations for polymyxin susceptibility testing as outlined in CLSI M100 and associated guidelines. In brief, colistin sulfate (Santa Cruz Biotechnology, TX, USA) was incorporated into Mueller–Hinton agar (Oxoid, UK) at a concentration of 3 µg/mL [28]. The prepared agar plates were stored at 4 °C and used within 48 h. Bacterial suspensions were adjusted to a 0.5 McFarland standard and subsequently diluted 1:10. A volume of 10 µL of each diluted inoculum was spot-inoculated onto the agar surface, with up to ten isolates applied per Petri dish. Plates were incubated at 35 °C for 16–20 h. Colistin resistance was defined by the growth of one or more colonies, corresponding to a minimum inhibitory concentration (MIC) >3 µg/mL. Quality control was performed using *Escherichia coli* ATCC 25922 [29]. All resistant isolates were preserved at −80°C in 20% glycerol stocks (Merck, Catalog #1.04094). Complete protocols, media formulations, and strain metadata are detailed in S1 Data and illustrated in Figures S1-S11 in S1 File, ensuring full methodological transparency and reproducibility.

### 2.4. Total DNA extraction and Metagenomic (16S rRNA) Sequencing

Two different DNA extraction kits were used following the manufacturer’s protocols to extract the total DNA from all 27 raw samples for metagenomics sequencing. The DNeasy PowerSoil Pro Kit (Qiagen, Germany; Cat. No./ ID: 47014) was used to process 3 feed samples and 6 sediment samples, while the QIAamp Fast DNA Stool Mini Kit (Qiagen, Germany; Cat. No./ ID: 51604) was employed for 6 chicken gut samples, 6 droppings samples, and 6 fish intestine samples (S1 Table). The extracted total DNA was subsequently processed for metagenomic sequencing. To amplify the V3-V4 region of the bacterial 16S rRNA gene, sequencing libraries were constructed using primer pairs 341F (5′-CCTACGGGNGGCWGCAG-3′) and 806R (5′-GACTACHVGGGTATCTAATCC-3′) that included the Illumina overhang adapter sequence (Illumina, Inc., San Diego, CA, USA). Each PCR reaction contained 12.5 uL of 2X KAPA HiFi HotStart Ready Mix (Roche Diagnostic Corporation, Indianapolis, IN, USA; Cat. No./ ID: 50-196-5299), 5 µl of DNA extract, 2.5 µl of 5 µM forward primer, 2.5 µl of 5 µM reverse primer, and 2.5 µl of HyClone water (Cytiva Life Sciences, Marlborough, MA, USA; Cat. No./ ID: SH30538.03). The cycling conditions were as follows: 95 °C for 3 min for initial denaturation, followed by 25 cycles of 95 °C for 30 s, 62 °C for 30 s, 72 °C for 30 s, with a final extension of 72 °C for 4 min. PCR amplifications were conducted using a T100TM Thermal Cycler (Bio-Rad Laboratories, Inc., Hercules, CA, USA; Cat. No./ ID: 1861096) and amplified products were visualized using a Fragment Analyzer™ Automated CE System (Agilent Technologies, Santa Clara, CA, USA; USA; Cat. No./ ID: M5311AA). The PCR products were purified using an epMotion 5075 Liquid Handler (Eppendorf AG, Hamburg, Germany; Cat. No./ ID: 5075006022) and AMPure XP beads (Beckman Coulter, Inc., Brea, CA, USA; Cat. No./ ID: A63882). The index PCR was performed using Illumina Nextera XT Index kit (Illumina, Inc., San Diego, CA, USA; Cat. No./ ID: 15032354) and the cycling conditions were as follows: 95 °C for 3 min for initial denaturation, followed by 25 cycles of 95 °C for 30 s, 56 °C for 30 s, 72 °C for 30 s, with a final extension of 72 °C for 4 min. The indexed libraries were purified using AMPure XP beads, normalized, pooled and the final QC was done using both Fragment Analyzer and Lightcycler 480 RT-PCR system (Roche Diagnostic Corporation, Indianapolis, IN, USA; Cat. No./ ID: 05015243001). The final pool was sequenced on an Illumina NextSeq2000 using a v3 600 cycle kit and P1 flow cell (Illumina, Inc., San Diego, CA, USA). The Georgia Genomics and Bioinformatics Core (GGBC) at the University of Georgia, USA handled the entire library preparation and sequencing process.

### 2.5 Taxonomic profiling of amplicon sequences using Qiime2 Pipeline

The sequencing of 27 samples was performed using Illumina paired-end technology (2 × 300 bp) [30,31] to ensure comprehensive coverage of the 16S rRNA V3-V4 hypervariable region. The 465 bp amplicon size corresponds to the V3-V4 hypervariable region of the 16S rRNA gene, amplified using primers 341F and 806R. This region was selected based on: (a) its established taxonomic resolution for microbial community studies [7] (b) compatibility with Illumina NextSeq2000 paired-end sequencing (2 × 300 bp); and (c) coverage of ~90% of bacterial taxa in the Greengenes2 database. The distribution of sequence lengths showed considerable consistency, with the interquartile range (25th to 75th percentiles) spanning from 443 bp to 465 bp, and the 2nd and 98th percentiles at 439 bp and 466 bp, respectively. This narrow distribution confirmed the high specificity of the amplification and the robustness of the bioinformatics pipeline. Initial quality assessment of FASTQ files was conducted using FastQC v0.12.1. Subsequent quality filtering and adapter trimming were carried out with Trimmomatic v0.39 [32] employing a sliding window of 30 bp, minimum read length cutoff of 100 bp, and minimum average quality score of 20. After quality control, samples yielded an average of 767,128 read pairs (range: 468,135−1,071,120), demonstrating sufficient sequencing depth for robust analysis (Table S1). Downstream processing was performed in QIIME 2 v2023.5 [33], where the VSEARCH algorithm implemented read joining, dereplication, and de novo Operational Taxonomic Unit (OTU) clustering at 99% sequence similarity with chimera removal. Taxonomic classification was achieved using the Greengenes2 v2022.10 database [34] through a trained naive Bayes classifier [31] with the classify-sklearn algorithm assigning taxonomy to OTUs. This pipeline ensured standardized processing while maintaining compatibility with established microbiome analysis protocols [35].

### 2.6. In silico functional genome analysis

Functional profiling of microbiome samples was performed using PICRUSt2 (Phylogenetic Investigation of Communities by Reconstruction of Unobserved States, v2.4.2) [36] with reference to the Kyoto Encyclopedia of Genes and Genomes (KEGG) Orthology database [37]. The analysis pipeline incorporated several quality control measures: (a) phylogenetic placement using SEPP (Skeletal Engine for Phylogenetic Placement) with Hidden State Prediction (HSP) method set to maximum posterior probability (mp) for optimal alignment accuracy; (b) a stringent minimum alignment score threshold of 0.8; and (c) a maximum Nearest Sequenced Taxon Index (NSTI) cutoff of 2.0 to exclude poor-quality predictions. The analysis was executed with verbosity mode enabled for comprehensive output logging, using an edge exponent parameter of 0 to normalize the influence of sequence placement on functional predictions. Resulting KEGG Orthology (KO) identifiers were mapped to specific metabolic pathways using the ggpicrust2 R package (v1.0.0) [38], followed by hierarchical classification of pathways into functional categories (Level 1) and subcategories (Levels 2–3) according to the KEGG BRITE hierarchy system [39]. This approach enabled systematic evaluation of microbial community functions while maintaining compatibility with established metagenomic analysis frameworks [40].

### 2.7. Statistical analysis and data visualization

Downstream analysis, including alpha and beta diversity assessments, microbial composition, and statistical comparisons, was performed using the “Phyloseq” package [41] in R software (version 4.4.1) (R Studio, 2021). OTU counts were normalized through rarefaction. Alpha diversity metrics including observed richness, Chao1, Shannon, and Inverse Simpson Diversity Index (InvSimpson) indices were estimated using the R packages “Vegan” [42], “ggpubr” [43], and “ggplot2” [44] and subsequently visualized to assess the community-level diversity patterns. Differences in microbial abundance and diversity between locations were identified using the Wilcoxon rank sum through test via the “microbiomeutilities” [45] R package. Upon obtaining a significant result from the Kruskal-Wallis test (p < 0.05), post-hoc pairwise comparisons were conducted using the Wilcoxon Rank-Sum test (Mann-Whitney U test) [46]. This test determines if one sample type has consistently higher or lower diversity values than another. The resulting p-values from all pairwise comparisons were then adjusted using the Benjamini-Hochberg method [47]. Beta diversity was evaluated through principal coordinate analysis (PCoA) based on Bray-Curtis dissimilarity [48], with differences between samples assessed using Permutational Multivariate Analysis of Variance (PERMANOVA) and permutation tests [49]. Taxonomic comparisons, heatmaps, and histograms were generated with the “Vegan,” “PHyloseq,” “microbiomeutilities,” “Tidyr” [50] and “ggplot2” packages. Statistical analyses were conducted in triplicate, and results were presented as the average of these three measurements. Additionally, computational (dry lab) counts were derived from 16S rRNA gene sequencing data processed using QIIME2 with downstream analysis performed in Excel 2016 (Microsoft Inc.). Taxonomic assignments were based on OTU clustering, with read counts aggregated by farm, sample type, and genus. Percentages represent the proportion of total classified reads. Merged categories were used where species-level resolution was not possible, enabling comparative insight between culture-based detection and high-throughput sequencing of the broader microbial community. This analysis provides a culture-independent perspective on microbial community structure, serving as a complementary approach to the targeted culturable isolate data generated through the wet lab workflow.

## 3. Results

### 3.1. Microbial community composition across sample types in integrated aquaculture systems

**Fig 1:**
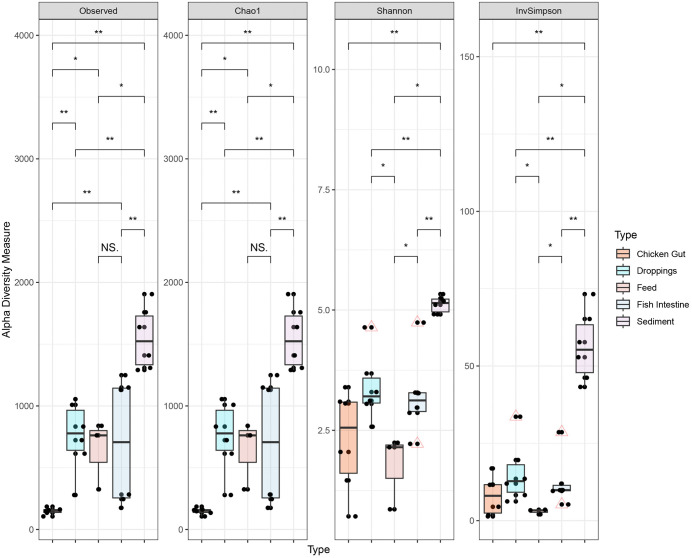
Microbial Alpha Diversity Analysis of Studied Communities. Alpha diversity, a measure of within-sample microbial richness and evenness, was evaluated across sample types using four complementary indices: observed richness, Chao1 (estimating total richness), Shannon, and Inverse Simpson. The distribution of these metrics is presented in boxplots with overlaid individual data points. Pairwise comparisons between sample types were performed using the Wilcoxon rank-sum test. Statistical significance is denoted on the figures as follows: ***p < 0.001, **p < 0.01, *p < 0.05; “n.s.” indicates non-significant differences. The x-axis represents the sample types, while the y-axis corresponds to the value of each alpha diversity index.

**Fig 2:**
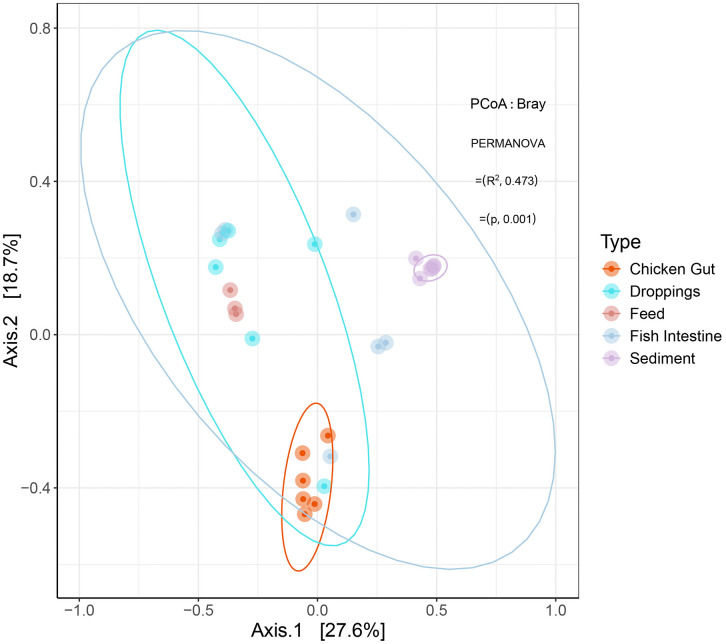
Microbial Beta Diversity Across Sample Types. Principal Coordinate Analysis (PCoA) based on Bray–Curtis dissimilarity illustrates distinct clustering of microbial communities according to sample type. PERMANOVA confirms that sample type explains a significant portion of the observed variation in community structure (R² = 0.473, p = 0.001). Each point represents a sample, colored by type, and the analysis was performed with 999 permutations.

We conducted a comprehensive analysis of within-sample (alpha) diversity to determine the principal factors influencing microbial community structure within integrated aquaculture systems. Our investigation, encompassing five distinct ecological niches, Chicken Gut, Droppings, Feed, Fish Intestine, and Sediment, revealed that sample type is the paramount determinant of microbial richness and diversity. The Kruskal-Wallis test, a non-parametric statistical method appropriate for this non-normally distributed data, identified highly significant overall differences among sample types for all major indices: observed richness (H = 20.9, p = 0.0003), estimated richness (Chao1, H = 20.9, p = 0.0003), Shannon diversity (H = 19.4, p = 0.0006), and Inverse Simpson diversity (H = 19.3, p = 0.0007) where H denotes the Kruskal–Wallis test statistic. Post-hoc pairwise comparisons using the Wilcoxon rank-sum test with Benjamini-Hochberg correction for multiple testing delineated the specific hierarchy of diversity across niches (Fig 1; Table 2). Sediment samples formed a distinct high-diversity cluster, exhibiting significantly greater richness (mean Chao1 ± SE = 1742.5 ± 128.5) and diversity (mean Shannon = 5.21 ± 0.05; mean Inverse Simpson = 59.2 ± 7.8) than all other sample types (all pairwise p < 0.01). This finding underscores the role of sediment as a complex environmental reservoir sustaining highly diverse and evenly distributed microbial assemblages. Gut-associated and fecal samples (Fish Intestine and Droppings) occupied an intermediate position. While these communities were significantly less diverse than Sediment (p < 0.01), they maintained substantially higher richness and diversity than Chicken gut and Feed samples. Notably, no significant difference in diversity was detected between Fish Intestine and Droppings communities (p > 0.05), suggesting a convergence in community structure between the gut lumen and its excreted byproducts in these systems. The most depauperate communities were consistently found in Feed and Chicken Gut samples. Feed, a processed and non-living substrate, supported the lowest Shannon diversity (1.75 ± 0.47), which was significantly lower than all other types (p < 0.05). Chicken Gut samples also showed remarkably low richness (148.8 ± 18.6) and diversity, which was statistically indistinguishable from Feed (p = 0.12) but significantly lower than both Fish Intestine (p < 0.05) and Droppings (p < 0.05). This indicates a strong selective filtering within the avian gut, resulting in a specialized, low-diversity microbiota, while the sterile nature of the feed provides a limited foundation for microbial colonization. To quantify the influence of sample type on overall microbial community composition (beta-diversity), we performed a permutational multivariate analysis of variance (PERMANOVA) based on Bray-Curtis dissimilarities. The analysis revealed that sample type explained a substantial and statistically significant portion of the variation in microbial community structure (PERMANOVA: R² = 0.474, F = 4.95, p = 0.001; 999 permutations) (Fig 2). This result indicates that nearly half (47.4%) of the observed variance in community composition is attributable to the ecological niche from which the sample was derived, establishing sample type as a primary determinant of microbial assemblage in this integrated aquaculture system. The highly significant p-value underscores the robustness of this finding against potential type I error. This profound separation suggests distinct, niche-specific microbial populations inhabit the Sediment, Droppings, Fish Intestine, Chicken Gut, and Feed environments, each shaped by vastly different ecological filters and selective pressures.

**Table 2:** Summary of alpha diversity metrics (mean ± standard error) by sample type.

Sample Type	N	Observed Richness	Chao1 Index	Shannon Index	Inverse Simpson
Sediment	6	1635.2 ± 85.9	1742.5 ± 128.5	5.21 ± 0.05	59.2 ± 7.8
Droppings	6	753.8 ± 138.2	832.5 ± 176.9	3.38 ± 0.56	14.4 ± 8.8
Fish Intestine	6	705.3 ± 320.8	771.8 ± 384.5	3.15 ± 0.58	10.7 ± 3.8
Chicken Gut	6	149.0 ± 15.6	148.8 ± 18.6	2.30 ± 0.46	7.9 ± 3.6
Feed	3	475.3 ± 252.3	642.0 ± 154.2	1.75 ± 0.47	3.0 ± 0.4

### 3.2. Distinct Phylum-Level Partitioning Reveals Niche-Specific Microbial Community Structures

**Fig 3:**
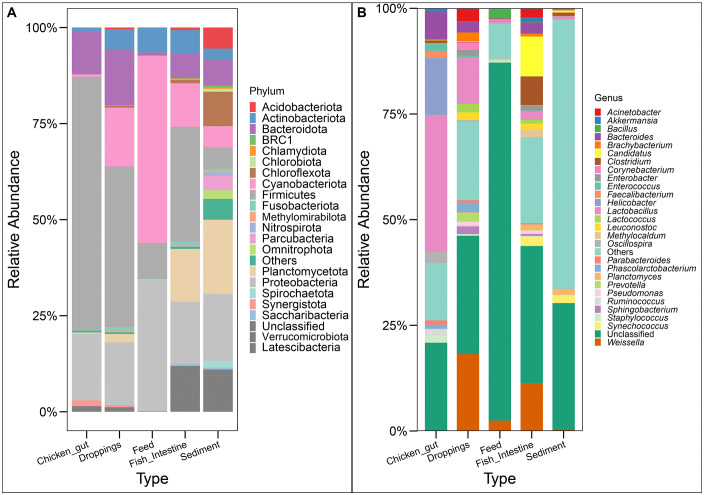
Microbial Community Composition at Phylum and Genus Level. Stacked bar charts display the relative abundance of the (A) top 22 microbial phyla and (B) top 28 microbial genera across all samples. Samples are grouped by type along the x-axis. The y-axis represents the percentage of sequences assigned to each taxon. Taxa are ordered from bottom to top by their mean relative abundance across the dataset.

Analysis of the relative abundance of bacterial phyla across the five sample types revealed profound and statistically significant differences in community structure, reinforcing that ecological niche is the primary determinant of microbial assemblage. A total of 65 phyla were identified across all samples, with the top 10 phyla comprising over 90% of the total sequences, consistent with typical microbial community patterns in environmental and host-associated systems (Fig 3A; Data S2). The composition of dominant phyla varied dramatically between sample types (Kruskal-Wallis, p < 0.001 for all major phyla). Firmicutes overwhelmingly dominated the chicken gut microbiota (mean relative abundance ± SE = 84.7% ± 3.2%), significantly exceeding levels in all other sample types (Wilcoxon rank-sum, p < 0.001 for all pairwise comparisons). This pattern is characteristic of the avian gastrointestinal tract, where Firmicutes typically prevail. Conversely, sediment samples displayed the most phylum-level diversity, with no single phylum exceeding 25% relative abundance, but were particularly enriched in Proteobacteria (23.0% ± 2.1%), Planctomycetota (17.1% ± 2.8%), and Verrucomicrobiota (11.7% ± 1.9%), all of which were significantly more abundant in sediment than in other sample types (p < 0.01). Notable gradient patterns were observed for several phyla. Bacteroidota abundance was highest in droppings (15.0% ± 2.5%) and chicken gut (14.8% ± 2.1%), intermediate in fish intestine (8.9% ± 1.7%), and lowest in sediment (3.5% ± 0.8%) and feed (0.3% ± 0.1%), with these differences being statistically significant (H = 18.3, p = 0.001). Cyanobacteria displayed an inverse pattern, dominating feed samples (67.2% ± 5.4%) but representing a minor component (<3%) of chicken gut communities (p < 0.001). Planctomycetota and Verrucomicrobiota showed remarkable specificity, being overwhelmingly associated with sediment environments (17.1% and 11.7% respectively) compared to minimal presence in feed and chicken gut samples (<0.5%; p < 0.001). This distribution suggests that these phyla comprise specialized oligotrophic taxa adapted to the sediment microenvironment. The Proteobacteria, a metabolically diverse phylum, were relatively abundant across all sample types but showed significantly higher representation in sediment (23.0% ± 2.1%) and feed (46.6% ± 4.8%) compared to animal-associated samples (15–17%; p < 0.05). This pattern suggests that different classes within this phylum have adapted to both free-living and host-associated lifestyles.

The significant phylum-level partitioning observed via PERMANOVA (R² = 0.474, p = 0.001) demonstrates that each ecological niche maintains a highly distinct phylogenetic signature, reflecting strong environmental filtering. Animal gut-associated samples (Chicken Gut and Fish Intestine) were predominantly structured by the phyla Firmicutes and Bacteroidota, a classic signature of vertebrate gastrointestinal tracts where these taxa are essential for energy harvest and host metabolism. In stark contrast, Feed samples were overwhelmingly dominated by Cyanobacteria, indicative of a primary production-based source such as spirulina or other cyanobacterial meal components, alongside a substantial population of Proteobacteria, likely representing environmental contaminants or opportunistic colonizers of the nutrient-rich substrate. The most complex and phylogenetically diverse communities were found in Sediment, which did not exhibit single-phylum dominance but was instead significantly enriched with Planctomycetota and Verrucomicrobiota, phyla known for their roles in nutrient cycling in aquatic environments, particularly nitrogen and sulfur metabolism. Furthermore, sediment served as the primary reservoir for a multitude of rare candidate phyla (Omnitrophota, Latescibacteria, and Parcubacteria), which collectively contributed to its status as the most diverse niche. This clear phylum-level stratification underscores that the microbial community in this integrated system is not homogenized but rather compartmentalized into discrete, niche-specific assemblages.

### 3.3. Genus-level taxonomic profiling reveals profound niche specialization in integrated aquaculture systems

Analysis of genus-level relative abundances revealed a complex and highly stratified microbial landscape across the five sample types, with community composition being predominantly structured by ecological niche (PERMANOVA: R² = 0.474, p = 0.001). A Kruskal-Wallis test confirmed that the relative abundance of the majority of dominant genera varied significantly across sample types (p < 0.01 after Benjamini-Hochberg correction), underscoring the powerful selective pressure imposed by each environment. Post-hoc pairwise Wilcoxon rank-sum tests delineated a clear hierarchy of niche specificity. Sediment communities exhibited the highest phylogenetic diversity and were uniquely enriched with genera involved in sulfur, nitrogen, and carbon cycling. Notably, sediment hosted significantly higher abundances of *Syntrophobacter* (1.20%, p < 0.001), *Methanosaeta* (1.13%, p < 0.001), and *Nitrospira* (0.50%, p < 0.01) compared to all other sample types, reflecting its anaerobic and chemolithotrophic conditions (Fig 3B, Data S2). Genera within the Planctomycetota phylum, which were nearly absent elsewhere, were prominent sediment residents. In stark contrast, Chicken Gut samples were dominated by a consortium of Firmicutes. The genus *Glycomyces* was overwhelmingly abundant (4.05%, p < 0.001) and specific to this niche, alongside significant enrichments of *Rummeliibacillus* (2.89%, p < 0.01), *Thermoactinomyces* (2.21%, p < 0.01), and *Sciscionella* (1.70%, p < 0.01) compared to all other types. This community profile indicates a specialized environment optimized for carbohydrate metabolism and host digestion. Fish Intestine samples displayed a hybrid signature, sharing some host-associated features with chicken gut but also showing influences from the aquatic environment and diet. While also rich in Firmicutes, the fish gut was uniquely and significantly enriched (p < 0.01) with Verrucomicrobia, particularly the genus *Akkermansia* (listed here within Verrucomicrobia at 11.63%), a mucin-degrader commonly associated with vertebrate guts. Feed samples were characterized by a strikingly different profile, dominated by photosynthetic Cyanobacteria (48.77%), a signature that was significantly higher than in any other sample type (p < 0.001). The genus *Oligella*, a Proteobacterium, was also a significant marker for feed (0.42%, p < 0.05). Droppings served as a transitional reservoir, sharing features with both the chicken gut and the external environment. While still rich in Firmicutes and Bacteroidetes from the gut, droppings showed the highest relative abundance of the genus*AF12* (0.24%, p < 0.05), within the Bacteroidetes. Wilcoxon pairwise comparisons confirmed that each sample type possesses a unique genus-level fingerprint. Sediment is a reservoir for biogeochemical cycling specialists, chicken gut is dominated by carbohydrate-processing Firmicutes, fish intestine presents a blend of host-associated and aquatic bacteria, feed is defined by photosynthetic cyanobacteria, and droppings represent a mixed community reflecting both gut origin and environmental exposure. This precise taxonomic resolution underscores the profound impact of local environmental conditions in shaping distinct microbial assemblages.

### 3.4. A Comparative analysis of wet lab culturing and computational metagenomics reveals divergent taxonomic profiles

A direct comparison of the outputs from experimental (wet lab) culture-based methods and computational (dry lab) 16S rRNA amplicon sequencing revealed fundamentally different profiles of microbial prevalence and diversity, highlighting the complementary nature of these approaches (Table 3). Both methodologies captured a comparable distribution of samples across the three farms. Farm 1, 2, and 3 were represented by 41.8%, 18.2%, and 40.0% of wet lab isolates, and 31.6%, 34.3%, and 34.2% of dry lab sequences, respectively. This indicates a balanced sampling strategy across the farms that was effectively captured by both techniques. The distribution of sample types (Feed, Chicken Gut, Droppings, Fish Intestine, and Sediment) was also consistent between the two analyses. The most striking finding was the extreme discrepancy in the organisms detected by each method. The culture-based approach was heavily biased toward a narrow spectrum of fast-growing, facultative anaerobic bacteria. Just three genera, *Escherichia* (25.5%), *Proteus* (25.5%), and *Staphylococcus* (14.5%), accounted for 65.5% of all isolated organisms. This method successfully cultured specific, often clinically relevant pathogens such as *Vibrio cholerae*, *Salmonella spp.*, and *Shigella flexneri*, which are primary targets for traditional microbiology. However, it failed to capture the vast majority of the microbial community, as evidenced by the complete absence of isolates from dominant phyla like Firmicutes (other than pathogens) and Bacteroidota that were prevalent in the molecular data.

**Table 3:** Organism Counts and Percentages from Experimental (Wet Lab) and Computational (Dry Lab) Analyses.

Experimental (Wet Lab)				Computational (Dry Lab)		
**Farm**	**Type**	**Organism Count**	**Percentage (%)**	**Type**	**Organism Count**	**Percentage (%)**
	Farm1	23	41.82	Farm1	5422380	31.57
	Farm2	10	18.18	Farm2	5882547	34.25
	Farm3	22	40.00	Farm3	5872548	34.19
**Samples**	Feed	4	7.27	Feed	1666513	9.24
	Chicken Gut	13	23.64	Chicken Gut	3606788	19.99
	Droppings	19	34.55	Droppings	3864313	21.42
	Fish Intestine	9	16.36	Fish Intestine	4077611	22.60
	Sediments	10	18.18	Sediment	4825142	26.75
**Organisms**	*Bacillus cereus*	1	1.82	*Bacillus*	57623	21.32
	*Escherichia coli*	14	25.45	*Escherichia*	27	0.01
	*Proteus mirabilis*	14	25.45	*Proteus*	106	0.04
	*Vibrio parahaemolyticus*	5	9.09	*Vibrio*	32	0.01
	*Staphylococcus aureus*	8	14.55	*Staphylococcus*	89467	33.11
	*Pseudomonas aeruginosa*	4	7.27	*Pseudomonas*	67958	25.15
	*Shigella flexneri*	1	1.82	*Shigella*	34285	12.69
	*Vibrio cholerae*	3	5.45	*Merged	N/Ap	N/Ap
	*Citrobacter spp.*	1	1.82	*Citrobacter*	20758	7.68
	*Mammaliicoccus spp.*	1	1.82	**Not Found**	N/A	N/A
	*Salmonella spp.*	3	5.45	*Salmonella*	8	0.002

In stark contrast, the computational metagenomic approach revealed a vastly complex and diverse microbial ecosystem. The analysis was dominated by genera that are typically underrepresented in culture, such as *Bacillus* (21.3%), *Staphylococcus* (33.1%), and *Pseudomonas* (25.1%). Notably, the genera that dominated the wet lab results (*Escherichia, Proteus, Vibrio*) were nearly absent in the metagenomic data, representing less than 0.06% of the total sequences. This suggests that these culturable organisms, while present, are minor components of the overall microbial community in these environmental and gut samples.

### 3.5. Culture-based isolation reveals diverse bacterial taxa with widespread antimicrobial resistance

A total of 55 bacterial isolates were recovered and analyzed from the integrated aquaculture system, representing a range of presumptive species. The most abundant isolates were *Escherichia coli* and *Proteus mirabilis*, each comprising 14 isolates (25.45% of the total). Other frequently observed species included *Staphylococcus aureus* (8 isolates, 14.55%), *Vibrio parahaemolyticus* (5 isolates, 9.09%), and *Pseudomonas aeruginosa* (4 isolates, 7.27%). Less commonly recovered taxa included Vibrio cholerae (3 isolates, 5.45%) and *Salmonella* spp. (3 isolates, 5.45%). Several species were detected only once, including *Bacillus cereus, Citrobacter* spp., *Shigella flexneri*, and *Mammaliicoccus* spp. (each 1 isolate, 1.82%).

**Fig 4:**
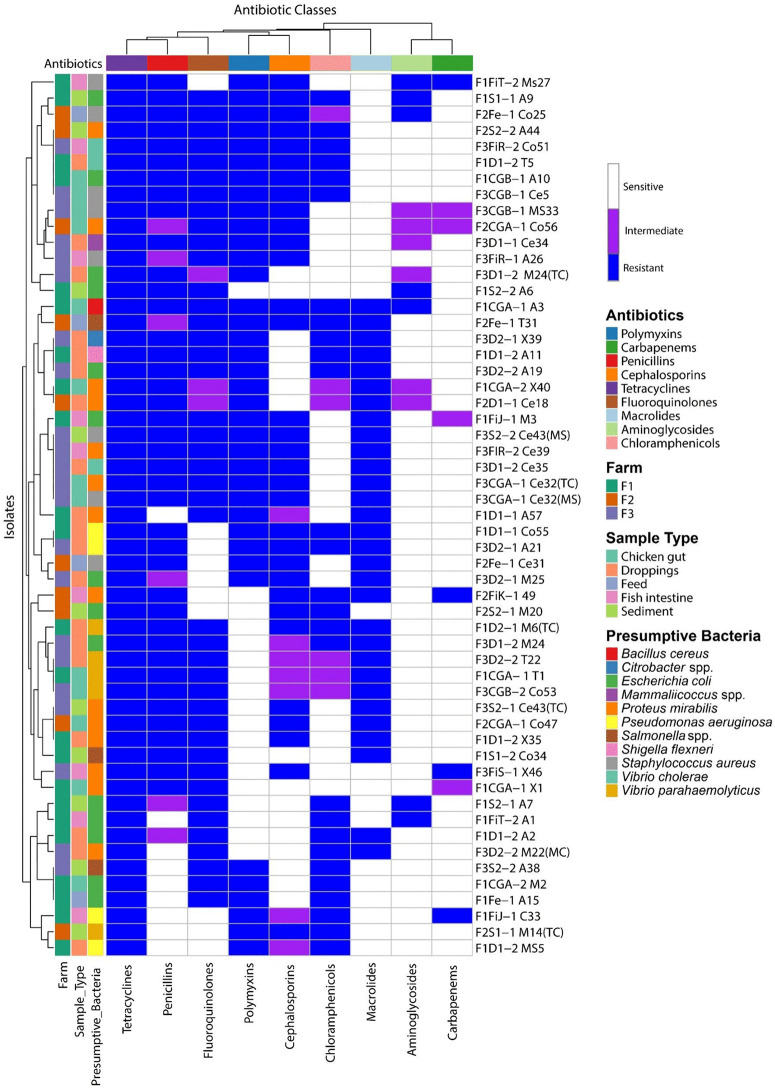
Antibiotic Resistance Profiles. This figure presents the antibiotic resistance profiles of bacterial isolates, highlighting high resistance levels to various antibiotic classes such as Tetracyclines, Fluoroquinolones, and Penicillins. The results underscore the prevalence of antibiotic-resistant strains like *Escherichia coli* and *Proteus mirabilis*, raising concerns about antibiotic use and resistance in integrated fish farming.

Subsequently, the antimicrobial resistance profiles of these isolates were evaluated, considering their origin from distinct ecological compartments within the integrated aquaculture system. Across different sample types, resistance was highest in droppings (33.94%), followed by chicken gut (23.72%), sediment (17.52%), fish intestines (17.16%), and feed (7.66%). Intermediate resistance (%I) was most notable in droppings (40.62%) and chicken gut (40.62%), while susceptibility (%S) was relatively higher in fish intestine (16.41%) and feed (6.88%). The resistance profile across antibiotic classes revealed that Tetracyclines had the highest resistance (20.07%), followed by Fluoroquinolones (15.33%) and Penicillins (14.60%). Polymyxins (13.50%) and Cephalosporins (11.68%) also showed considerable resistance. Moderate resistance was observed against Macrolides (10.95%) and Chloramphenicols (9.85%), while Aminoglycosides (2.56%) and Carbapenems (1.46%) displayed the lowest rates of resistance. Intermediate resistance was most pronounced for Cephalosporins (21.88%), Chloramphenicols (18.75%), and Aminoglycosides (18.75%). Among the bacterial species, *E. coli* exhibited the highest resistance (23.72%), with significant intermediate resistance (21.87%) and moderate susceptibility (28.57%). *P. mirabilis* showed similar trends, with 24.45% resistance, 34.38% intermediate, and 25.40% susceptibility. *S. aureus* demonstrated 16.06% resistance, while *V. parahaemolyticus* exhibited 8.03% resistance but high intermediate levels (18.75%). *P. aeruginosa* contributed 6.93% resistance, while *V. cholerae* (6.57%), *Salmonella* spp. (5.11%), *B. cereus* (2.92%), *Citrobacter* spp. (2.19%), *S. flexneri* (2.20%), and *Mammaliicoccus* spp. (1.82%) each contributed modestly to the resistance burden. These findings indicate a concerning distribution of antibiotic resistance among common bacterial pathogens, particularly *E. coli* and *P. mirabilis*, which remain the major contributors. Intermediate resistance was widespread across multiple classes, underscoring the emergence of reduced susceptibility that may complicate treatment strategies (Fig 4).

### 3.6. Functional Genomic Insights via KEGG Orthology Mapping

**Fig 5:**
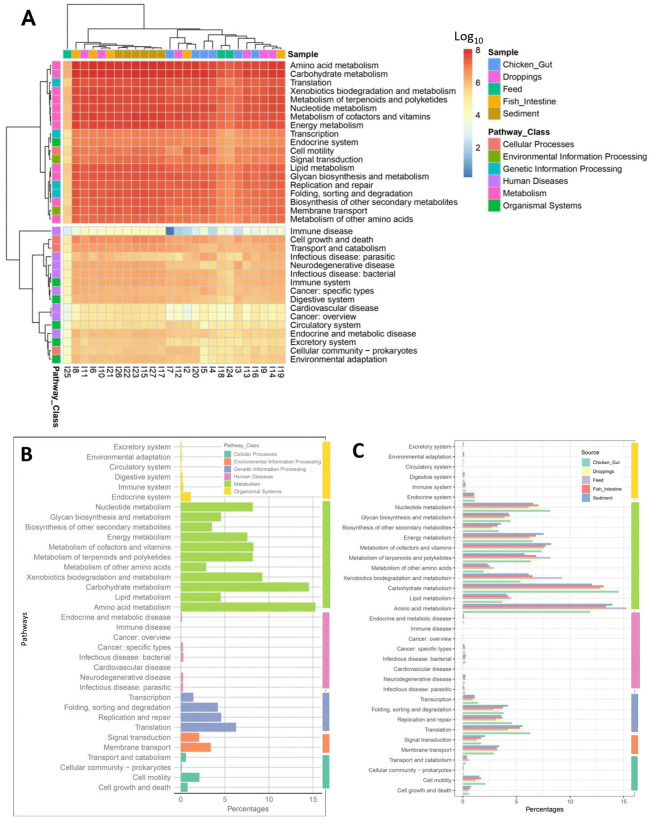
Distribution of Pathway Enrichment Across Samples. Fig 5A presents a clustering analysis of KEGG pathways, revealing distinct patterns among the samples (Feed, Chicken Gut, Droppings, Fish Intestine, and Sediment), with amino acid and carbohydrate metabolism pathways enriched in Feed, while translation and replication pathways are more prominent in Chicken Gut and Droppings. Fig 5B indicates that metabolism pathways are the most abundant, particularly amino acid (67.56%) and carbohydrate metabolism (65.70%). In contrast, human diseases and organismal systems pathways are less dominant**.** Fig 5C highlights that Feed exhibits elevated metabolic pathways, especially amino acid metabolism (15.27%), while Sediment shows greater representation of environmental and genetic processing pathways.

The clustering of different pathways shows clear distinctions between the samples (Feed, Chicken Gut, Droppings, Fish Intestine, and Sediment) (Fig 5A). Pathways related to metabolism, such as amino acid and carbohydrate metabolism, appear more enriched in Feed, while pathways like translation and replication are more prominent in Chicken Gut and Droppings. Sediment shows higher clustering in environmental information processing pathways.

The pathways involved in Metabolism are the most highly represented across the samples (Fig 5B). Specifically, Amino acid metabolism (67.56%) and Carbohydrate metabolism (65.70%) dominate, indicating their significant roles in the microbial communities. Other notable metabolism-related pathways include Xenobiotics biodegradation and metabolism (34.04%), Metabolism of terpenoids and polyketides (34.14%), and Energy metabolism (33.57%), all contributing to the functional diversity observed. Pathways related to Genetic Information Processing also show high abundance, with Translation (26.98%) and Replication and repair (18.97%) being prevalent. In contrast, pathways under Cellular Processes such as Cell motility (8.09%) and Environmental Information Processing like Membrane transport (15.60%) and Signal transduction (8.35%) exhibit moderate representation. Additionally, Human Diseases and Organismal Systems pathways are less dominant, with the highest percentages observed for neurodegenerative disease (1.10%) and Immune system (1.27%), respectively. This distribution highlights the metabolic and genetic functional capacity of the samples.

The abundance of each pathway is distributed across the five samples. Metabolism pathways, especially amino acid metabolism, are consistently higher in all samples, particularly in Feed (15.27%) (Fig 5C). Other pathways, such as membrane transport, are more evenly distributed, with Sediment showing a slightly higher abundance (3.41%) compared to the other samples. Overall, Sediment tends to have higher representation in environmental and genetic processing pathways, while Feed is more dominant in metabolic pathways.

Table 4 highlights key pathways involved in Xenobiotics biodegradation and Metabolism within an integrated farming system. Notably, Drug metabolism – cytochrome P450 showed a high presence in Chicken gut (23.27%), while Drug metabolism – other enzymes was notably abundant in Sediment (34.87%), and Bisphenol degradation reached its highest in Droppings (30.18%). Fluorobenzoate degradation was prominent in Fish intestine (32.37%), and Toluene degradation stood out in Feed (11.71%). Additional pathways of interest include Aminobenzoate degradation, with its highest representation in Sediment (34.76%), and Naphthalene degradation, which was abundant across Fish intestine (28.62%) and Sediment (28.38%). Metabolism of xenobiotics by cytochrome P450 also demonstrated elevated levels in Fish intestine (29.38%) and Sediment (28.94%), suggesting active detoxification processes. Polycyclic aromatic hydrocarbon degradation and Styrene degradation were relatively high across samples, underscoring their widespread roles in xenobiotic processing within the farming system.

**Table 4:** Xenobiotics Biodegradation and Metabolism Pathways Across Samples.

Xenobiotics biodegradation and Metabolism Pathways	Feed (%)	Chicken Gut (%)	Droppings (%)	Fish Intestine (%)	Sediment (%)
Nitrotoluene degradation	3.80	16.18	23.05	26.35	30.61
Styrene degradation	4.31	15.59	24.41	26.99	28.70
Aminobenzoate degradation	3.65	15.80	20.12	25.67	34.76
Drug metabolism – cytochrome P450	2.60	23.27*	24.19	25.48	24.47
Benzoate degradation	5.19	15.72	27.64	24.93	26.52
Drug metabolism – other enzymes	3.57	11.10	19.64	30.81	34.87*
Polycyclic aromatic hydrocarbon degradation	4.42	15.66	24.27	27.48	28.15
Naphthalene degradation	5.65	12.78	24.57	28.62	28.38
Xylene degradation	3.23	17.15	21.92	25.66	32.04
Chloroalkane and chloroalkene degradation	4.42	17.12	25.40	26.06	27.01
Metabolism of xenobiotics by cytochrome P450	3.49	11.30	26.90	29.38	28.94
Caprolactam degradation	3.37	16.95	25.24	29.03	25.41
Atrazine degradation	4.00	14.97	25.20	27.27	28.55
Ethylbenzene degradation	7.17	17.63	27.95	24.05	23.19
Chlorocyclohexane and chlorobenzene degradation	5.21	16.11	23.26	29.06	26.37
Bisphenol degradation	7.56	10.99	30.18*	28.92	22.36
Dioxin degradation	4.50	20.85	29.38	27.20	18.07
Fluorobenzoate degradation	5.34	4.14	27.39	32.37*	30.76
Toluene degradation	11.71*	4.01	23.21	30.49	30.57

### 3.7. Correlation Analysis Between Microbial Genera and Antibiotic Resistance Genes Identified through KEGG Pathways

The analysis of antibiotic resistance pathways identified through KEGG Orthology reveals significant insights into genes associated with antibiotic resistance and their prevalence in various samples. Beta-lactamase resistance emerged as a prominent feature across the sampled environments. The gene *bcrC*, encoding undecaprenyl-diphosphatase, was highly abundant in sediment (58.99%) and chicken gut samples (31.03%), with notable presence also detected in droppings (34.99%) and fish intestines (29.23%). Additionally, *acrA, mexA, adeI, smeD, mtrC*, and *cmeA* genes were notably abundant in fish intestine (25.74%) and sediment samples (6.58%). Vancomycin resistance was observed, primarily mediated by the *vanX* gene encoding zinc D-Ala-D-Ala dipeptidase, with significant abundances in sediment (10.92%) and droppings (12.07%). Least abundant genes *oprM, emhC, ttgC, cusC, adeK, smeF, mtrE,* and *cmeC,* were found in chicken gut (7.18%) and sediment samples (1.40%) (Data S3).

The Spearman correlation analysis of microbial co-occurrence patterns revealed distinct relationships among bacterial genera across different sample types (Data S3). Strong positive correlations (ρ = 1) were particularly evident in gut-associated samples, with *Helicobacter* and *Lactobacillus* showing complete correlation (ρ = 1) in chicken gut and intestinal samples. This likely reflects their symbiotic relationship in the gastrointestinal tract. Similarly, *Oscillospira* and *Lactobacillus* demonstrated perfect correlation (ρ = 1) in droppings and gut samples, suggesting their coordinated role in digestion and nutrient metabolism. The feed samples showed unique correlation patterns, with *Acinetobacter* exhibiting strong positive associations (ρ = 1) with *Leuconostoc* and *Brachybacterium*, possibly indicating microbial interactions in feed digestion. In sediment samples, we observed significant positive correlations (ρ = 0.974) between *Dechloromonas* and *Methylosinus*, along with their associations with *Synechococcus* and *Geobacter*, suggesting potential redox-coupled metabolic interactions in the aquatic environment. Notably, fish intestine samples displayed strong positive correlations (ρ = 1) among *Ruminococcus*, *Enterococcus*, and *Faecalibacterium,* consistent with their known roles as core gut microbiota. These patterns were less pronounced in other sample types, highlighting habitat-specific microbial relationships. The feed and sediment samples showed particularly strong negative correlations (ρ = −0.974 to −1), with *Dechloromonas* and *Staphylococcus* exhibiting near-perfect negative correlation (ρ = −0.974) in sediments, possibly reflecting niche competition or antagonism. Sample-type specific patterns emerged clearly, with gut and intestinal samples showing more numerous and stronger positive correlations compared to environmental samples. The metadata analysis revealed that these correlation patterns were maintained across different farms despite geographical variations, suggesting robust microbial interactions within each sample type. The strongest negative correlations (ρ = −1) were observed between *Megamonas* and *Synechococcus* in fish intestine samples, potentially indicating habitat exclusion between gut-associated and free-living bacteria. These results demonstrate that microbial co-occurrence patterns are strongly influenced by sample type, with gut-associated samples showing more cooperative interactions and environmental samples exhibiting more competitive relationships. The consistency of these patterns across different farm locations suggests that these microbial interactions are driven by habitat characteristics rather than geographical factors.

**Fig 6:**
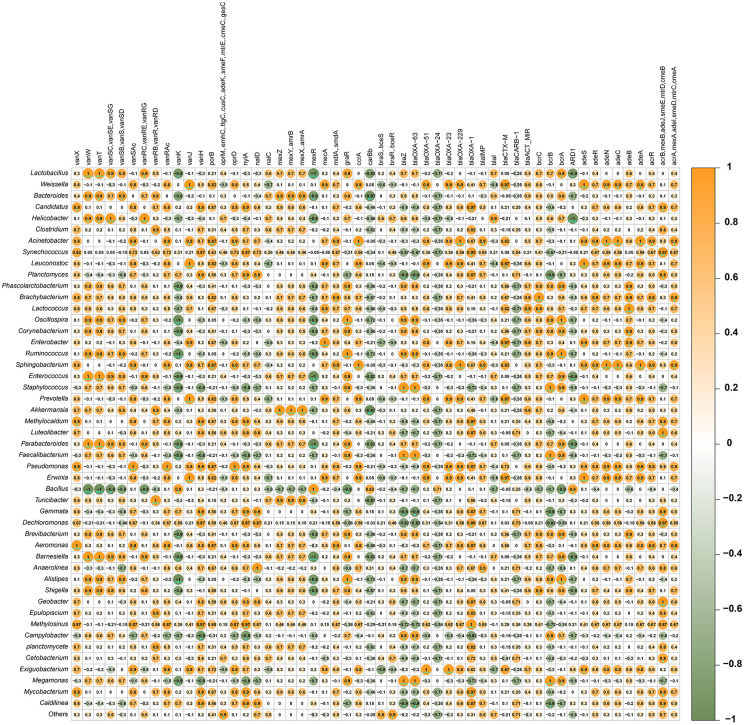
Antibiotics resistance genes (ARGs) detected different samples and their Spearman’s correlation with top 50 dominant microbial genera. This figure presents a correlation matrix, a visual representation of the relationships between top 50 dominant bacterial genera (listed on the left) and a set of AMR genes (listed across the top). The numbers display the Spearman’s correlation coefficient **(r)**. Vivid Orange and Moss Green indicate positive and negative correlation, respectively. The intensity of the color, along with the size of the circles, reflected the strength of these relationships, where larger and darker circles denoted stronger correlations. *Significant level (*p < 0.05; **p < 0.01; ***p < 0.001).

The correlation matrix, a comprehensive visualization of the relationships between bacterial genera and specific genes, reveals a complex network of associations. Notably, antibiotic resistance genes exhibit diverse correlation patterns across bacterial taxa, suggesting variations in their dissemination and functional impacts. For instance, the *blaOXA* family of genes, including *blaOXA-1*, *blaOXA-23*, *blaOXA-24*, *blaOXA-51*, and *blaOXA-63*, as well as *blaIMP*, displayed a range of correlations with genera such as *Acinetobacter*, *Pseudomonas*, and *Bacteroides*, indicating potential differences in their prevalence and activity within these bacterial communities. Similarly, the *acrB, mexB* efflux pump gene, associated with multidrug resistance, showed both positive and negative correlations with various genera, including *Pseudomonas*, *Acinetobacter*, and *Enterobacter*, suggesting context-dependent roles in different bacterial species. The *van* operon genes, responsible for vancomycin resistance, including *vanX*, *vanW*, *vanT*, *vanSC*, *vanSB*, *vanS*, *vanR*, *vanK*, *vanJ*, and *vanH*, also demonstrated varied correlations with genera like *Enterococcus* and *Clostridium*, potentially reflecting different resistance mechanisms or regulatory pathways. Beyond antibiotic resistance, other genes, such as *nylA*, *nalD*, *nalC*, *mexZ*, *mexY*, *amrB*, *braS*, *boeS*, *braR*, *boeR*, *blaZ*, *moxR*, *mecA*, *mdlA*, *smdA*, *graR*, *corA*, *carB*, *berC*, *berB*, *berA* and genes involved in cell wall synthesis and transport (*oprM*, *emhC*, *llgC*, *cusC*, *adeK*, *smeF*, *mtrE*, *omeC*, *gesC*), exhibited distinct correlation profiles across various genera. *Lactobacillus* and *Pseudomonas* showed markedly different correlation patterns, highlighting the functional divergence between these genera. *Lactobacillus* displayed unique correlations with genes like *vanX* and *vanW*, while *Pseudomonas* showed distinct patterns with *blaOXA* genes and *acrB, mexB*. Genera such as *Bacteroides*, *Clostridium*, and *Enterococcus* also exhibited unique correlation signatures. While some genes, such as *acrB, mexB*, showed broad correlations across genera like *Pseudomonas*, *Acinetobacter*, and *Enterobacter*, suggesting conserved functions, others displayed genus-specific associations, indicating tailored adaptations. For instance, *mecA* showed a strong positive correlation with *Staphylococcus*, while *nylA* showed a distinct correlation pattern with *Acinetobacter* (Fig 6).

## 4. Discussion

### 4.1. Overview of taxonomic and ecological patterns in integrated aquaculture microbiota

The taxonomic analysis of microbial communities revealed a significant predominance of bacteria over archaea—a pattern consistent with many environmental microbiomes, where bacterial diversity often surpasses that of archaea [51]. Among the bacterial phyla, Firmicutes were dominant, aligning with their known roles in fermentation and gut health, particularly in avian species [52]. Their high abundance in chicken gut samples underscores their essential role in digestion and nutrient absorption, corroborating previous findings that highlight the importance of Firmicutes in avian gut microbiota [53].

Cyanobacteria were notably more abundant in feed samples, likely due to the feed composition that includes materials rich in cyanobacterial biomass [54]. The varying distribution of Proteobacteria across sample types reflects their ecological versatility and role in nutrient cycling [55]. Unique archaeal genera, such as Haloarchaea, were identified in feed samples, indicating niche-specific adaptation [56]. In contrast, the minimal presence of archaea in chicken gut samples supports findings that certain gut environments are less favorable for archaeal colonization [57].

At the genus level, the high prevalence of *Lactobacillus* in chicken gut samples highlights its vital role in avian gut health, including enhancing digestion and immune response [58]. The significant presence of *Weissella* in droppings suggests its involvement in lactic acid fermentation and potential probiotic functions [59]. *Helicobacter*, found specifically in chicken gut samples, aligns with its known pathogenic role in avian gastrointestinal systems [60]. Other genera such as *Candidatus*, *Bacteroides*, and *Clostridium* were detected across different environments, reflecting their involvement in nutrient cycling and host-microbe interactions [61]. The detection of unique bacterial genera in sediment and the recurrence of certain genera across sample types suggest potential microbial transfer between environmental sources and host organisms, supporting the concept of environmental reservoirs influencing gut microbiota composition [62].

### 4.2. Microbial diversity patterns

Alpha diversity analysis indicated significant differences in microbial richness and diversity across sample types. Sediment samples exhibited the highest diversity, consistent with their complex and heterogeneous nature [63]. Fish intestine samples displayed higher richness than feed, likely due to the variety in dietary inputs and environmental exposures inherent in integrated aquaculture systems [64]. Conversely, chicken gut and droppings had lower richness, reflecting selective pressures that favor specific gut-associated taxa, mainly *Lactobacillus* and *Weissella* [65]. Beta diversity analysis further confirmed the distinct microbial community compositions among sample types. Sediment samples clustered separately, indicating unique microbial profiles [66]. Partial overlap of fish intestine samples with droppings and chicken gut suggests some shared taxa, likely due to common diet and environmental exposure. These observations support the idea that both diet and host interactions shape microbial communities [67]. Significant group differences identified by PERMANOVA emphasize the role of environmental and host-related factors in structuring microbial diversity [68,69].

The observed patterns of microbial richness and diversity suggest that distinct ecological niches within the system harbor unique microbial communities, prompting an examination of the specific taxa that drive these differences across sample types. Feed samples were dominated by *Proteobacteria* and *Actinobacteria*, with *Weissella* and *Bacillus* genera common—indicating adaptation to nutrient-rich environments [70]. Chicken gut microbiota was enriched in *Firmicutes*, particularly *Lactobacillus*, essential for digestion and pathogen exclusion [53]. Droppings revealed a mix of *Firmicutes*, *Actinobacteria*, and *Proteobacteria*, alongside environmental genera such as *Acinetobacter* and *Corynebacterium*, suggesting complex interactions driven by habitat and diet [71,72]. In fish intestine samples, *Actinobacteria* and *Firmicutes* predominated, including *Weissella*, *Clostridium*, and *Candidatus*, reflecting their roles in aquatic nutrient cycling [73]. Sediment samples harbored a broad range of taxa, including unique genera like *Synechococcus* and *Planctomyces*, associated with photosynthesis and biogeochemical cycling [62,74].

These compositional distinctions are further underscored by statistically significant variations at both phylum and genus levels, highlighting the selective pressures and ecological adaptations that shape niche-specific microbial assemblages. Phyla such as Verrucomicrobia, Planctomycetes, and Proteobacteria varied significantly across environments, highlighting their adaptability. Verrucomicrobia, involved in polysaccharide degradation, and Planctomycetes, associated with nitrogen cycling, showed environment-specific distributions [75,76]. Genus-level differences were also notable: *Sphingobacterium* and *Synechococcus* (organic degradation and photosynthesis), *Staphylococcus* (pathogenicity), and *Weissella* (fermentation) were differentially abundant [59,77–79]. Opportunistic genera like *Enterococcus*, *Acinetobacter*, and *Corynebacterium* further illustrated adaptation across ecological niches [80].

### 4.3. Antibiotic resistance profiles

The study identified a concerning prevalence of antimicrobial resistance genes (ARGs) among common pathogens in the integrated farming system. High resistance rates in *Escherichia coli* and *Proteus mirabilis* reflect their role as reservoirs of resistance genes [81]. The predominance of resistance to tetracyclines, fluoroquinolones, and penicillin is consistent with their widespread agricultural and clinical use [82]. Lower resistance to aminoglycosides and carbapenems suggests these agents remain relatively effective, likely due to restricted use [83]. The detection of resistance in pathogens such as *Vibrio cholerae* and *Salmonella* spp. underscores the potential public health risks [84]. Variation in ARG prevalence across sample types reflects environmental exposure to antimicrobials. Elevated resistance in droppings and chicken gut likely stems from direct antibiotic administration, while resistance in sediment and fish intestine samples indicates environmental contamination [85,86]. Notably, the presumptive Gram-positive bacteria demonstrated consistent resistance to both polymyxins and cephalosporins, which corresponds to their well-established intrinsic resistance patterns and was identical to the results obtained with the control strains. This finding aligns with previous reports that Gram-positive organisms generally lack the outer membrane target of polymyxins, rendering them intrinsically resistant [11,87–89]. Similarly, their resistance to cephalosporins has been widely documented as a result of the presence of low-affinity penicillin-binding proteins and inherent structural barriers [90,91]. These consistent results support the reliability of our susceptibility testing outcomes.

### 4.4. Functional and metabolic profiling

KEGG-based functional analysis revealed a dominance of metabolic pathways across all samples. Genes associated with amino acid, carbohydrate, and energy metabolism are vital for microbial survival and adaptation [92]. Chicken gut samples were enriched with genetic information processing genes, indicating heightened transcriptional and translational activity [93]. Sediments harbored more environmental information processing genes, suggesting adaptive responses to changing conditions [94]. Pathways related to xenobiotic biodegradation, including cytochrome P450-mediated drug metabolism, were enriched in gut and sediment samples, illustrating microbial roles in detoxifying environmental pollutants [94,95]. These capabilities may support sustainability in farming by mitigating contamination.

### 4.5. ARG-microbiome correlation analysis

Correlation analysis revealed genus-specific associations with ARGs. Efflux pump genes (*acrB*, *mexB*) were strongly linked to *Pseudomonas*, *Acinetobacter*, and *Enterobacter* [96]. *Van* operon genes correlated with *Enterococcus* and *Clostridium*, indicating resistance to vancomycin [97]. *MecA*, a marker of methicillin resistance, was strongly associated with *Staphylococcus*, a concern in both human and veterinary medicine [98]. Notably, *Lactobacillus* correlated with specific vancomycin resistance genes (*vanX*, *vanW*), while *Pseudomonas* was linked to β-lactamase genes (*blaOXA*), highlighting divergent resistance profiles shaped by ecological niche and selective pressure [99]. These findings underscore the risk of ARG dissemination from environmental and gut microbiomes to pathogens, emphasizing the need for integrated surveillance and antibiotic stewardship to mitigate zoonotic transmission [14,100].

### 4.6. Study limitations and future directions

This study generated the first microbiota and ARG profiles of an integrated farming system, although it had a limited range of samples and environments as well as only partially understanding the mechanisms of origin of antibiotic resistance. Further work should include larger sampling campaigns, study these microbial communities in a longitudinal or temporal way, and expand this work with other techniques such as metatranscriptomics. Another limitation is that the concentrations of antibiotics have not been directly measured in the environmental samples or in the biological samples. An important area of research would be to measure the concentrations of antibiotics to better understand the relationship between antibiotic use and resistance development based on the presence of ARGs.

## 5. Conclusion

This study provides the first in-depth characterization of the microbiota and associated antimicrobial resistance from integrated chicken and fish farming systems in Bangladesh and identifies the ecological and human health risks posed by the environmental and gut-associated microbiota genes. The findings highlight the need for improved antimicrobial stewardship and optimal animal husbandry practices to reduce the spread of antimicrobial resistance genes to support sustainable nutrient cycling and safeguard human and animal health.

## Supporting information

S1 Data Provides detailed information on AST, isolates, biochemical tests and media used for culturing. (XLSX)

S2 Data Taxonomic profiling, metadata, sequence quality reports. (XLSX)

S3 Data KEGG Pathway abundance, percentages, resistance genes, and Ko numbers from the Picrust2 analysis. (XLSX)

S1 Table Sequencing and sampling metadata and quality reports. (DOCX)

S1 File Supplementary Figures. Bacterial Isolation, Biochemical Identification, Phyla Distribution, Antimicrobial Susceptibility Testing, and Colistin MIC Determination.
(DOCX)
