# Supplementary material for: Microbial diversity, functional genomics and antibiotic resistance in integrated chicken and fish farming systems of Bangladesh
Source: PLoS One. 2026 Apr 8;21(4):e0344367. doi: 10.1371/journal.pone.0344367 (PMC13061223; doi:10.1371/journal.pone.0344367)
Supplement: S1 Table — (DOCX) [file pone.0344367.s004.docx]

**Table S1:** 16S Metagenomic Sequencing Quality Assessment of Integrated Fish Farming System in Bangladesh.

| **Sample ID** | **SRA Accession Number** | **Input Read Pairs** | **Q20 %** | **Q30 %** | **PhiX** | **Instrument** | **Illumina run ID (Flowcell)** |
| --- | --- | --- | --- | --- | --- | --- | --- |
| I-1 | SRS22303601 | 764511 | 99.27 | 99.25 | 0 | VH00301 | AACM7N3M5 |
| I-2 | SRS22303600 | 778880 | 99.26 | 99.24 | 0 | VH00301 | AACM7N3M5 |
| I-3 | SRS22303612 | 471439 | 99.30 | 99.27 | 0 | VH00301 | AACM7N3M5 |
| I-4 | SRS22303620 | 689464 | 99.25 | 99.24 | 0 | VH00301 | AACM7N3M5 |
| I-5 | SRS22303621 | 717735 | 99.27 | 99.25 | 0 | VH00301 | AACM7N3M5 |
| I-6 | SRS22303622 | 694189 | 99.19 | 99.18 | 0 | VH00301 | AACM7N3M5 |
| I-7 | SRS22303623 | 838401 | 99.23 | 99.22 | 0 | VH00301 | AACM7N3M5 |
| I-8 | SRS22303625 | 803335 | 99.25 | 99.23 | 0 | VH00301 | AACM7N3M5 |
| I-9 | SRS22303624 | 681675 | 99.24 | 99.23 | 0 | VH00301 | AACM7N3M5 |
| I-10 | SRS22303626 | 922218 | 99.23 | 99.21 | 0 | VH00301 | AACM7N3M5 |
| I-11 | SRS22303602 | 830327 | 99.20 | 99.18 | 0 | VH00301 | AACM7N3M5 |
| I-12 | SRS22303603 | 774201 | 99.19 | 99.17 | 0 | VH00301 | AACM7N3M5 |
| I-13 | SRS22303604 | 670748 | 99.27 | 99.26 | 0 | VH00301 | AACM7N3M5 |
| I-14 | SRS22303605 | 694591 | 99.19 | 99.17 | 0 | VH00301 | AACM7N3M5 |
| I-15 | SRS22303606 | 1045042 | 99.15 | 99.13 | 0 | VH00301 | AACM7N3M5 |
| I-16 | SRS22303607 | 578786 | 99.27 | 99.26 | 0 | VH00301 | AACM7N3M5 |
| I-17 | SRS22303608 | 939844 | 99.14 | 99.12 | 0 | VH00301 | AACM7N3M5 |
| I-18 | SRS22303609 | 702670 | 99.34 | 99.32 | 0 | VH00301 | AACM7N3M5 |
| I-19 | SRS22303610 | 739951 | 99.24 | 99.22 | 0 | VH00301 | AACM7N3M5 |
| I-20 | SRS22303611 | 802329 | 99.27 | 99.26 | 0 | VH00301 | AACM7N3M5 |
| I-21 | SRS22303612 | 842458 | 99.25 | 99.24 | 0 | VH00301 | AACM7N3M5 |
| I-22 | SRS22303613 | 886374 | 99.15 | 99.13 | 0 | VH00301 | AACM7N3M5 |
| I-23 | SRS22303615 | 1079555 | 99.22 | 99.20 | 0 | VH00301 | AACM7N3M5 |
| I-24 | SRS22303617 | 629180 | 99.32 | 99.30 | 0 | VH00301 | AACM7N3M5 |
| I-25 | SRS22303616 | 506637 | 99.37 | 99.36 | 0 | VH00301 | AACM7N3M5 |
| I-26 | SRS22303618 | 799534 | 99.21 | 99.19 | 0 | VH00301 | AACM7N3M5 |
| I-27 | SRS22303619 | 988609 | 99.21 | 99.19 | 0 | VH00301 | AACM7N3M5 |
