# Supplementary material for: Microbial diversity, functional genomics and antibiotic resistance in integrated chicken and fish farming systems of Bangladesh
Source: PLoS One. 2026 Apr 8;21(4):e0344367. doi: 10.1371/journal.pone.0344367 (PMC13061223; doi:10.1371/journal.pone.0344367)
Supplement: S1 File — Bacterial Isolation, Biochemical Identification, Phyla Distribution, Antimicrobial Susceptibility Testing, and Colistin MIC Determination. (DOCX) [file pone.0344367.s005.docx]

**Biochemical Tests for Presumptive Isolate Identification**


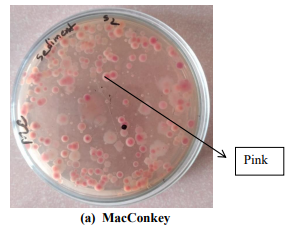


Fig. S1: Culturing samples on MacConkey agar


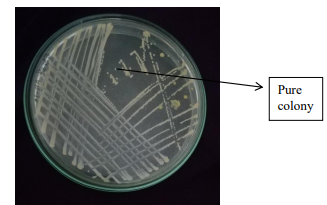


Fig. S2: Subculturing on Nutrient Agar


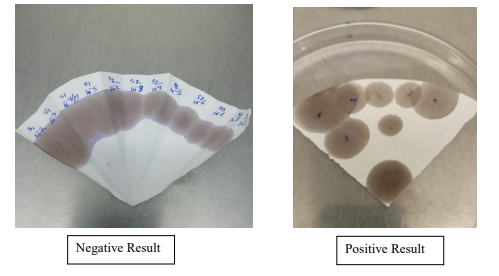


Fig. S3: Oxidase Test


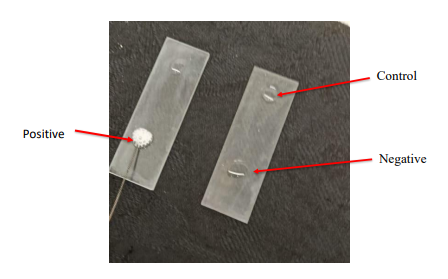


Fig. S4: Catalase Test


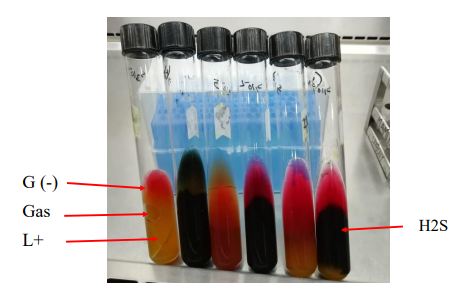


Fig. S5: KIA Test


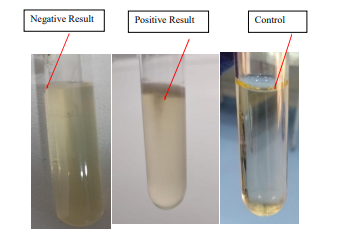


Fig. S6: Motility Test


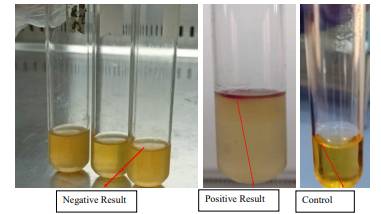


Fig. S7: Indole Test


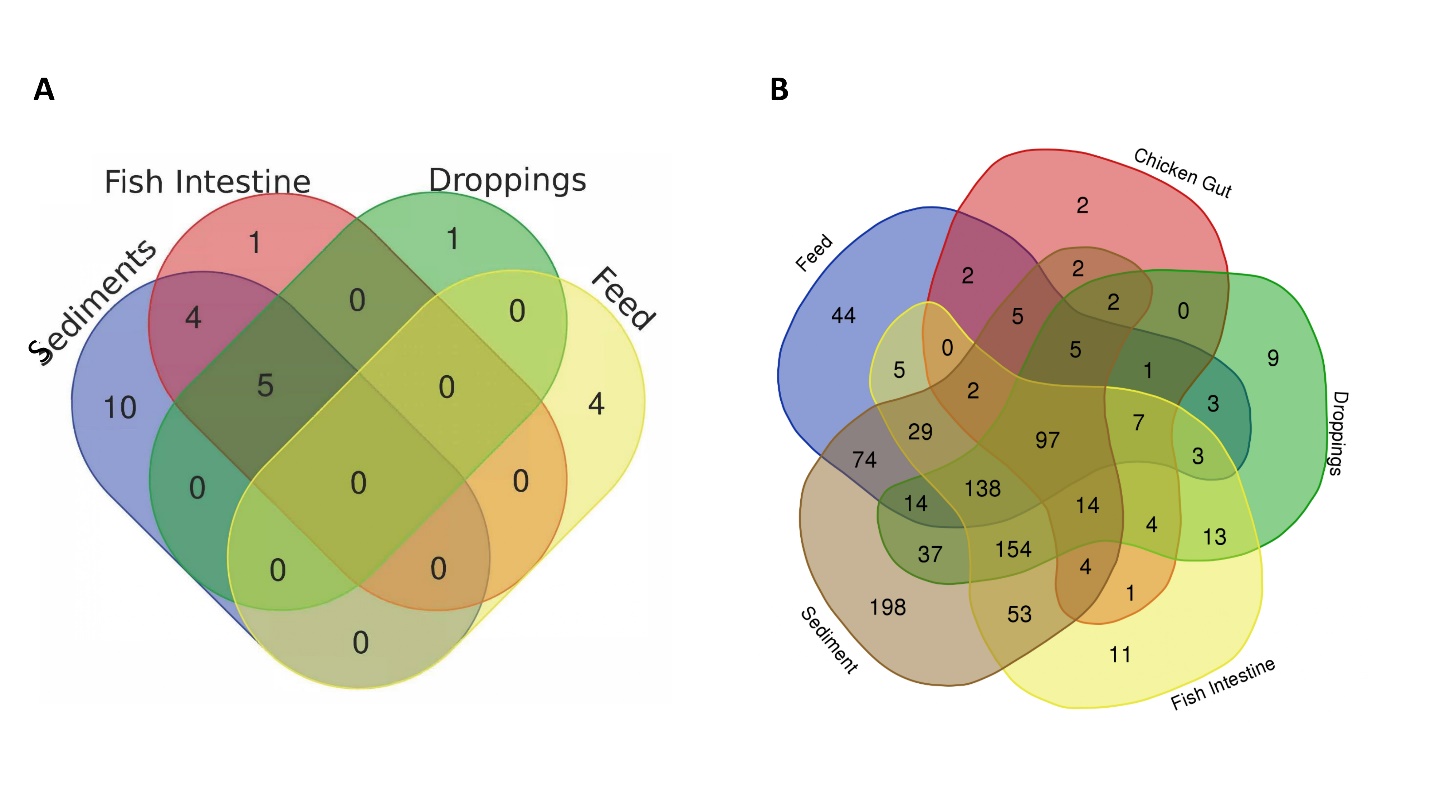


Fig S8: Distribution of shared and Unique Phyla among samples


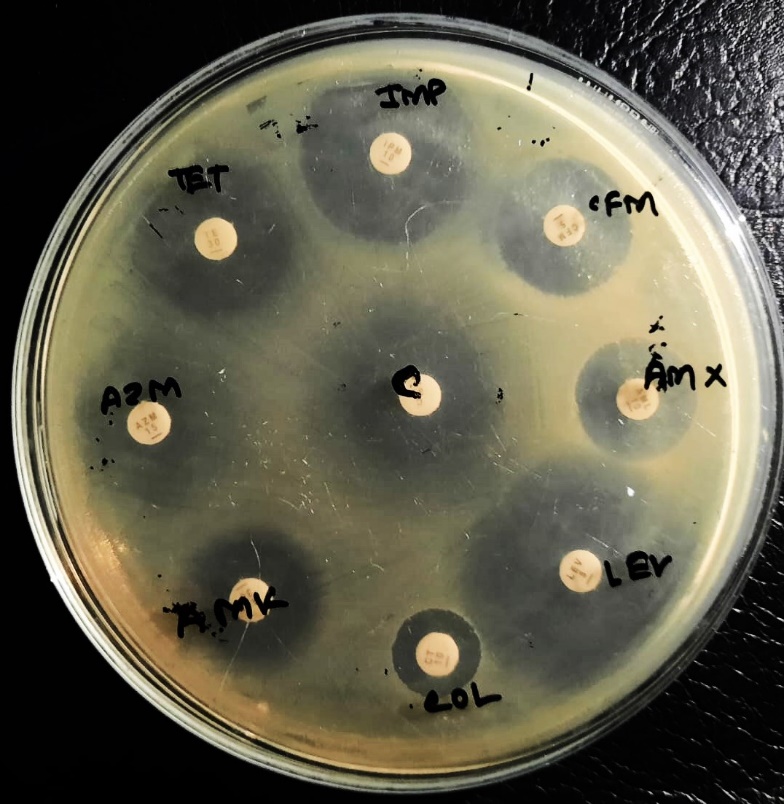


Fig S9: **Antimicrobial Susceptibility testing of *E. coli* ATCC 25922**

**
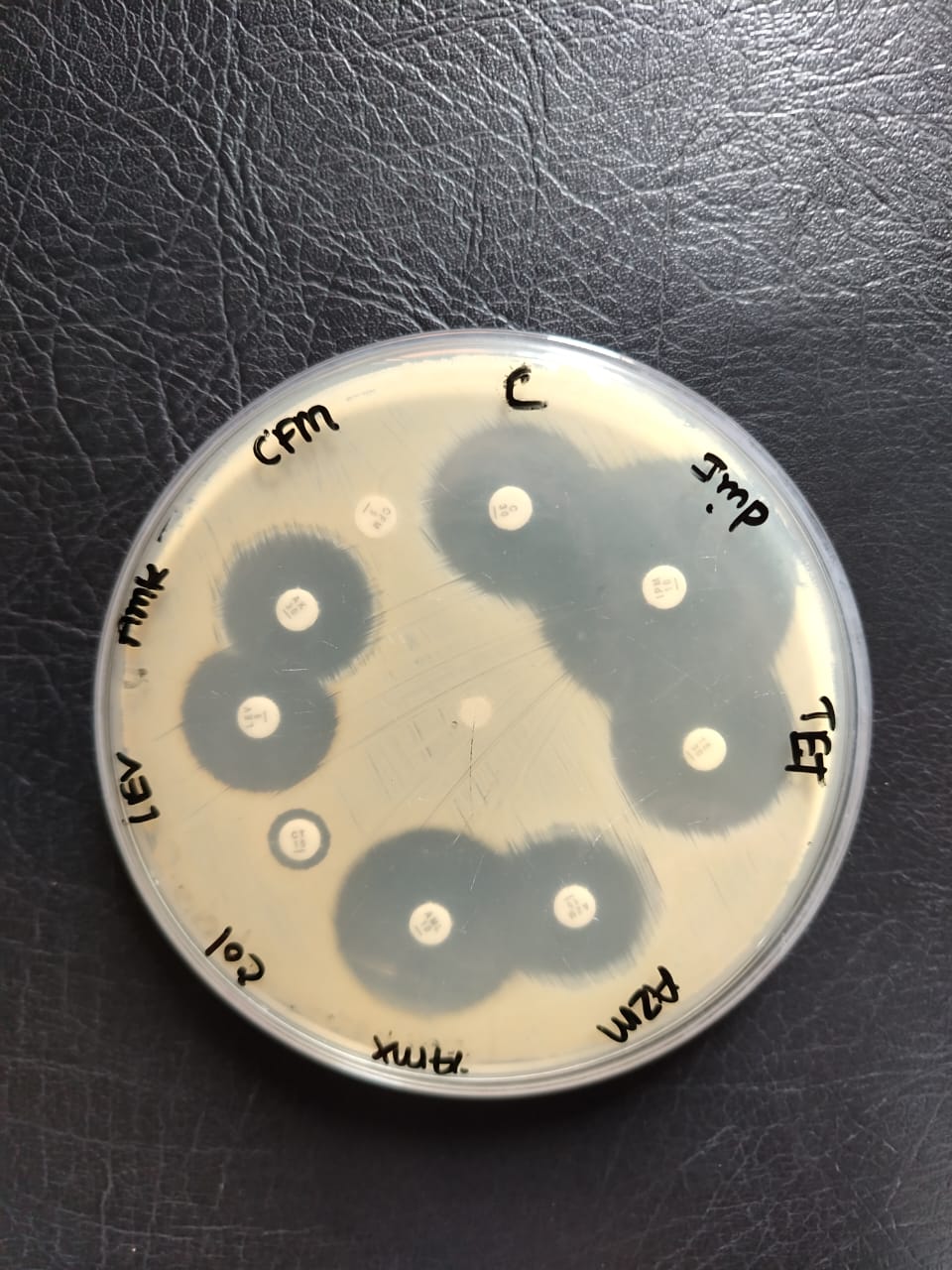
**

Fig S10: **Antimicrobial Susceptibility testing of *Staphylococcus aureus* ATCC 25923**

**
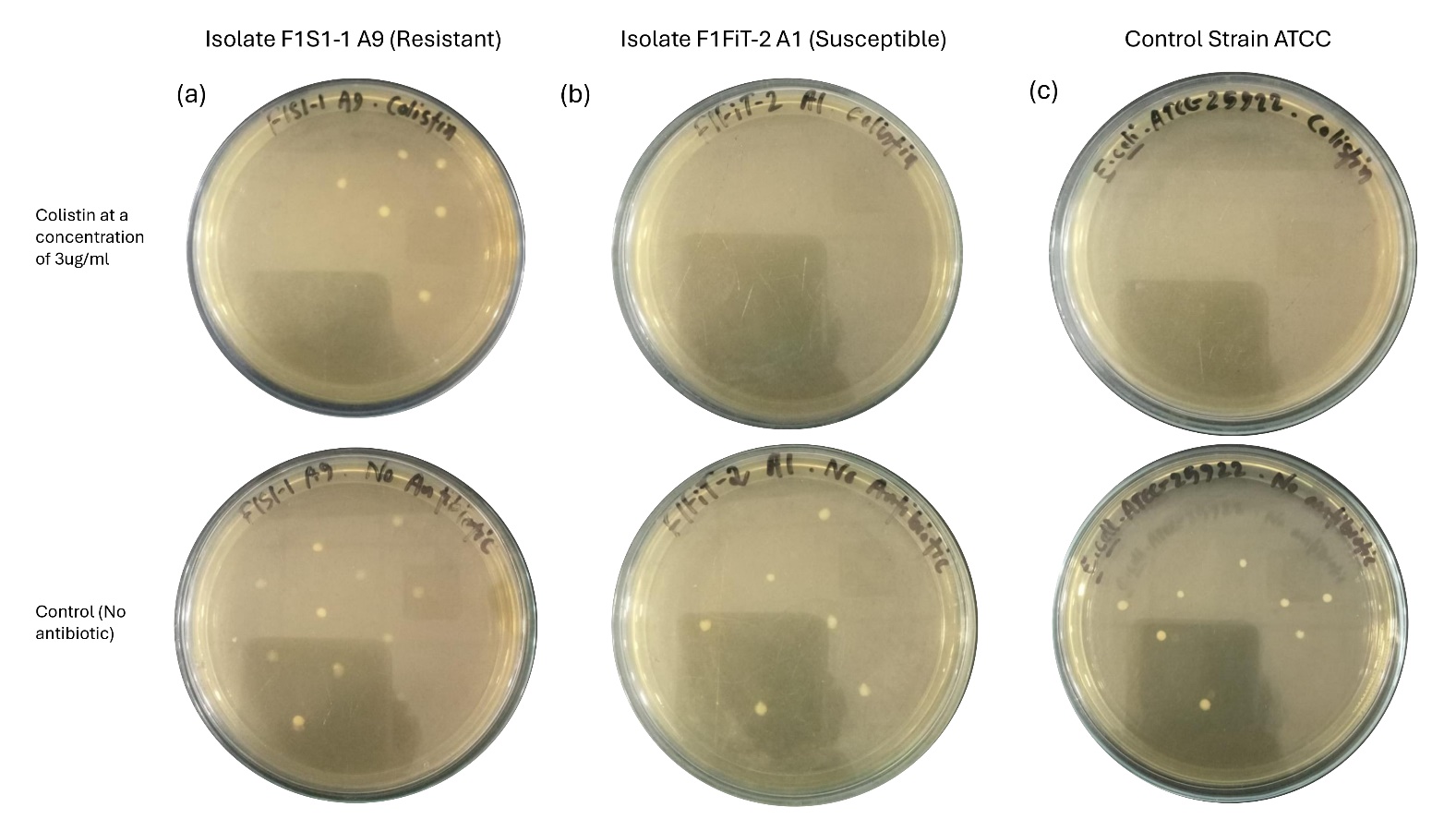
**

**Fig S11:** Agar dilution method for determination of colistin minimum inhibitory concentration (MIC). Mueller–Hinton agar plates supplemented with colistin (3 µg/mL) were used to assess colistin susceptibility of clinical isolates and quality control strains. (a) Growth of isolate F1S1-1 A9 on colistin-containing agar, indicating colistin resistance (MIC >3 µg/mL). (b) Absence of growth of isolate F1FiT-2 A1 on colistin-containing agar, indicating colistin susceptibility. (c) *Escherichia coli* ATCC 25922 showing no growth on colistin-containing agar as the quality control strain. The lower panels represent antibiotic-free Mueller–Hinton agar plates demonstrating growth of all isolates and the control strain, confirming inoculum viability.
